# Goutte de la hanche chez une femme de 57 ans: rapport de cas

**DOI:** 10.11604/pamj.2020.37.306.20859

**Published:** 2020-12-03

**Authors:** Dammak Nabil, Cheikhrouhou Hassen, Balti Walid, Khalifa Issam, Sghir Mouna, Kessomtini Wassia, Abid Faouzi

**Affiliations:** 1 Faculté de Médecine de Monastir, Service d’Orthopédie, CHU Taher Sfar, Mahdia, Tunisia,; 2 Faculté de Médecine de Monastir, Service de Médecine Physique, CHU Taher Sfar, Mahdia, Tunisia

**Keywords:** Goutte, obésité, tophus, imagerie, rapport de cas, Gout, obesity, tophus, imaging, case report

## Abstract

Cette étude rapporte le cas d'une arthrite goutteuse de la hanche, décrite chez une femme de 57 ans, diabétique et hypertendue, sans antécédents de goutte et qui a consulté pour douleur chronique de la hanche droite. L´examen clinique a trouvé une patiente obèse, marchant avec une boiterie droite douloureuse en rapport avec flessum de la hanche homolatérale. La radiographie standard a montré des formations ostéo-condensantes en regard de la face antérieure du col fémoral. La biologie se résume en un syndrome inflammatoire avec un taux normal d´acide urique. La tomodensitométrie et l'imagerie par résonance magnétique (IRM) nous ont orientés vers une chondromatose de la hanche. La patiente a eu un traitement chirurgical avec exérèse complète d´une formation friable et arthrolyse de la hanche. Les prélèvements bactériologiques n´ont pas isolé de germe et l´étude anatomopathologique a conclu à une goutte de la hanche. Un traitement médical à base de Colchicine a été introduit. L´évolution a été marquée par une régression complète du flessum, disparition de la boiterie et amélioration des douleurs de la hanche de façon satisfaisante.

## Abstract (en)

This study reports a case of gouty arthritis in the hip joint in a 57-year-old woman with diabetes and hypertension, with no history of gout, presenting with chronic pain in the right hip. Clinical examination showed obese patient, having painful right limping with lateral hip flexion during walking. Standard radiography objectified osteocondensing formations at the level of the anterior femoral neck. Laboratory tests showed inflammatory syndrome with normal uric acid concentration. Computerised tomography (CT) scan and magnetic resonance imaging (MRI) suggested chondromatosis of the hip. The patient underwent surgical treatment based on complete excision of the friable formation and hip arthrolysis. Bacteria were not isolated during the anatomopathological study which showed gout affecting the hip. The patient received medical treatment based on Colchicine. The patient had complete recovery of flexion, disappearance of limping and satisfactory improvement in hip pain.

## Introduction

La goutte, maladie métabolique associée à une arthropathie microcristalline, principalement périphérique et mono-articulaire, avec une prévalence significative chez les hommes âgés de 30 à 40 ans [1,2]. Bien que les lésions axiales dues à l'urate monosodique (MSU) soient considérées comme rares, elles semblent en réalité être sous-diagnostiquées [3,4]. L'insuffisance rénale, l'obésité, l'hypertension, les diurétiques et le salicylate (acide acétylsalicylique) sont des facteurs de risque de la goutte axiale [3]. La découverte de cristaux de MSU dans les aspirations articulaires est la marque de la goutte tophacée [5]; mais leur détection par microscopie polarisante peut être incohérente [1,6]. L'étude pathologique d'un échantillon de biopsie est une option invasive à effectuer dans des cas sélectionnés [3]. Les méthodes d'imagerie sont utiles pour montrer des lésions érosives avec un surplomb marginal, en plus du dépôt de microcristaux dans les articulations et les tissus adjacents, et des phénomènes inflammatoires locaux [1,2,4]. L'imagerie par résonance magnétique (IRM), la scintigraphie et la tomodensitométrie à double énergie (DECT) constituent des explorations qui peuvent confirmer les lésions goutteuses. L'IRM peut détecter le tophus, l'œdème et l'inflammation de la moelle osseuse, et le DECT permet de mieux détecter la présence et les effets de MSU dans les articulations [1,2,4]. Le but de ce manuscrit est de souligner, en s´aidant de la littérature, les critères cliniques, biologiques et radiologiques pouvant faire suspecter une goutte axiale, considérée comme maladie peu commune.

## Patient et observation

**Informations du patient:** une femme de 57 ans, diabétique et hypertendue sous traitement non hyperuricémiant, qui a consulté pour des douleurs récidivantes de la hanche droite, d´allure inflammatoire, sans notion de traumatisme, évoluant depuis 3 ans avec des épisodes aigues de blocage, ce qui l'empêchait de déambuler.

**Figure 1:**
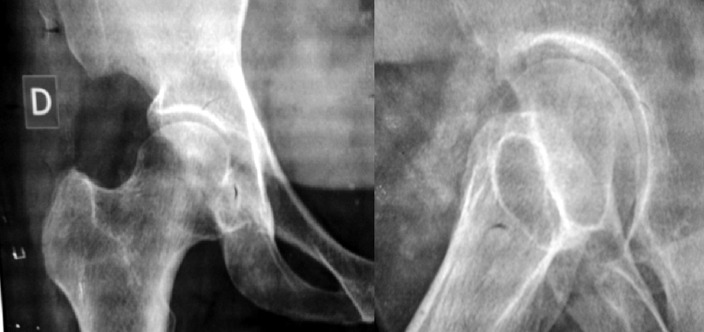
radiographies standards hanche droite face et profil montrant une formation dense sur le versant antérieur du col du fémur

**Figure 2:**
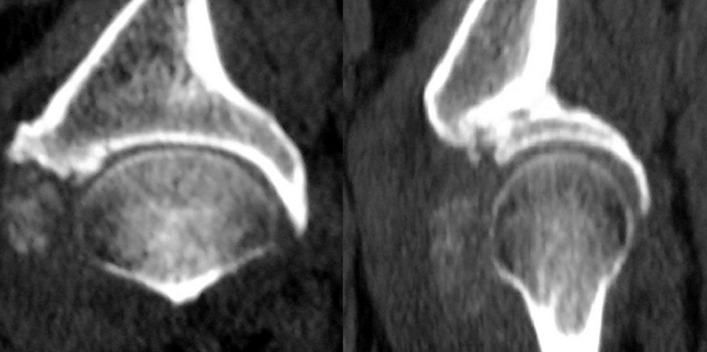
aspect scannographique de la formation

**Figure 3:**
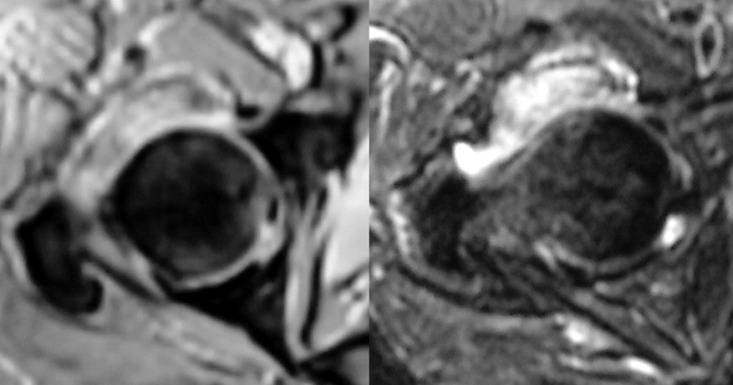
IRM montrant une formation en hyposignal T1 et hypersignal T2 fat sat

**Évaluation diagnostique:** l'examen physique a objectivé un flessum douloureux de la hanche droite de 20°, une douleur réveillée par la mobilisation de l'articulation coxofémorale, un indice de masse corporelle (IMC) 32 kg/m^2^ et un œdème modéré des deux membres inférieurs. Aucun tophus goutteux n'a été détecté. Le bilan radiologique standard du bassin de face et de la hanche droite de face et de profil, a mis en évidence une formation oblongue, faiblement dense, en regard de la face antérieure du col fémoral, suggérant une chondromatose articulaire (Figure 1). La biologie a montré un syndrome inflammatoire, avec des globules blancs à 11000 et une vitesse de sedimentation (VS) accélérée à 120/125. Cependant, le dosage de l´acide urique a été dans les normes. La tomodensitométrie a montré un aspect hyperdense de ces granulations, orientant aussi vers une chondromatose de la hanche (Figure 2). L´IRM a donné l´aspect de multiples formations ovalaires infra centimétriques formant des granulations en hyper signal T2 Fat Sat et hypo signal T1, siégeant au niveau du récessus antéro-supérieur de la cavité articulaire avec un œdème des tissus mous adjacents aux muscles fessiers et à la grosse tubérosité fémorale associé à une distension de la bourse trochantérienne (Figure 3).

**Intervention thérapeutique:** la patiente a été opérée à ciel ouvert par voie de Hardinge avec exérèse complète d´une formation friable, siégeant sur le versant antérieur de la hanche, suivie d´un lavage abondant de l´articulation et une synovectomie partielle. L´analyse microbiologique du liquide articulaire a été stérile. L´examen anatomopathologique a confirmé le diagnostic de goutte et un traitement médical à base de Colchicine a été instauré. L´évolution a été marquée par une nette amélioration de la mobilité de la hanche avec régression complète du flessum et récupération d´une marche autonome. Toutefois, bien que les douleurs de la hanche aient régressé d´une façon considérable, la patiente a présenté quelques épisodes algiques, très bien jugulés par des antalgiques et des anti-inflammatoires. Actuellement, soit à 4 ans de recul, la patiente garde une fonction satisfaisante.

## Discussion

Les épisodes de goutte aigus sont typiquement périphériques, mono-articulaires ou oligo-articulaires; cependant, dans la phase chronique de la maladie, l'atteinte peut devenir polyarticulaire [1,3,5,7]. Les tophi se développent plus souvent dans la goutte chronique, mais ils peuvent être le premier signe de la maladie [7]. Bien que la goutte axiale soit rare, l'insuffisance rénale chronique, l'obésité, l'hypertension artérielle, la présence de diurétiques et l'utilisation de salicylates à faible dose constituent les principaux facteurs de risque de cette entité [3]. Les modifications ostéoarticulaires sont dues aux cytokines proinflammatoires, aux chimiokines et aux enzymes dégradant la matrice, produites par les cellules inflammatoires et immunes [2]. Des outils d´imagerie radiographique, tels que la radiographie standard, la tomodensitométrie, la tomographie par ordinateur et la scintigraphie, peuvent montrer les cristaux MSU et les tophi qui peuvent contribuer aux lésions osseuses et articulaires de la goutte [2]. L'évaluation de 42 patients goutteux a montré une goutte axiale dans 29% des cas, des tophi axiaux dans 12% des cas, des érosions ou des calcifications dans 17% des cas. Les fréquences des lésions lombaires, thoraciques et sacro-iliaques étaient respectivement de 100%, 42% et 18% [4]. L'évaluation radiographique simple de 290 patients goutteux avec moins de 10 ans d'évolution de la maladie a fourni des preuves supplémentaires que le développement du tophus joue un rôle dans les érosions osseuses [8,9].

Une évaluation de l'œdème, des érosions, des tophi et des synovites a été réalisée par IRM chez 40 patients goutteux. Des érosions ont été trouvées dans 63% des cas et ont montré une association significative avec la présence de tophi; Cependant, il n'y avait pas de relation entre les érosions et la présence d'un œdème osseux [9]. La prévalence radiographique de l'arthropathie goutteuse sacro-iliaque est estimée entre 13% et 17%, et l'atteinte axiale dans son ensemble peut aller jusqu'à 35%; Cependant, deux principaux diagnostics différentiels à exclure sont l'arthrite infectieuse et l'ostéomyélite [3]. Cependant, le diagnostic d'arthrite infectieuse co-existante avec la goutte a été écarté dans le cas présent par les résultats de l´investigation microbiologique du liquide articulaire. Cependant, la possibilité que la goutte provoque des lésions imitant une infection a également été envisagée [7]. Les cristaux de MSU et les tophi jouent un rôle dans l'érosion osseuse et les lésions des articulations touchées par la goutte, mais ces changements peuvent se développer sans tophi à un stade avancé ou après des crises de goutte récurrentes [2].

## Conclusion

L'arthrite goutteuse de la hanche est considérée comme une affection très rare; mais elle semble être sous-diagnostiquée et/ou sous-rapportée. Elle pose un problème de diagnostic positif et différentiel avec les autres causes de douleur chronique de la hanche, surtout si elle constitue la seule manifestation de la maladie goutteuse. Les études de cas pourraient nous rappeler certaines spécificités de cette pathologie et savoir y suspecter devant certains critères radiologiques.
